# Long Working Hours and Risk of Venous Thromboembolism

**DOI:** 10.1097/EDE.0000000000000862

**Published:** 2018-07-31

**Authors:** Mika Kivimäki, Solja T. Nyberg, G. David Batty, Ida E. H. Madsen, Adam G. Tabák

**Affiliations:** 1 Department of Epidemiology and Public Health, University College London, London, United Kingdom; 2 Clinicum, Faculty of Medicine, University of Helsinki, Helsinki, Finland; 3 Finnish Institute of Occupational Health, Helsinki, Finland, m.kivimaki@ucl.ac.uk; Clinicum, Faculty of Medicine, University of Helsinki, Helsinki, Finland; Department of Epidemiology and Public Health, University College London, London, United Kingdom; National Research Centre for the Working Environment, Copenhagen, Denmark; 7 Department of Epidemiology and Public Health, University College London, London, United Kingdom; 8 Semmelweis University Faculty of Medicine, 1st Department of Medicine, Budapest, Hungary

## Abstract

Supplemental Digital Content is available in the text.

## To the Editor:

Venous thromboembolism (VTE) results from a blood clot that forms within a vein.^1^ It includes two subtypes: deep-vein thrombosis (a clot in a deepvein, usually in the leg) and pulmonary embolism (a sudden blockage in a lung artery). Studies of people sleeping in deck chairs in air-raid shelters during the second world war and, more recently, those of passengers on long-haul flights have linked extended periods of sitting to increased VTE risk.^2^ It is also the case that psychological stress can unfavorably influence blood coagulation and viscosity, potentially increasing the risk of VTE.^3,4^ People working long hours are often characterized by both sedentary behavior and stress, but to our knowledge, no studies are available on the association of this working pattern with VTE. This is therefore the focus of the present analyses.

We drew individual-level data from eight prospective cohort studies participating in the Individual–Participant–Data meta-analysis in Working Populations (“IPD-Work”) Consortium.^5^ We excluded people not in full-time employment and those with extant disease at study baseline. Working hours and participant characteristics were assessed at baseline (total N = 77,005 to 77,291 depending on the outcome; eAppendix; http://links.lww.com/EDE/B359). All study members were followed up for VTE for a mean of 9.7 years.

As previously,^6–8^ we defined ≥55 hours/week as long working hours, with a standard working week of 35–40 hours representing the reference category. Incident VTE was ascertained using linkage to electronic records for hospitalizations and deaths in national registers. We defined VTE using *International Classification of Disease* diagnostic codes (Table). During 830,550 person-years at risk, 539 VTE events were recorded: 350 with deep-vein thrombosis and 258 with pulmonary embolism (69 participants had both).

**TABLE.**
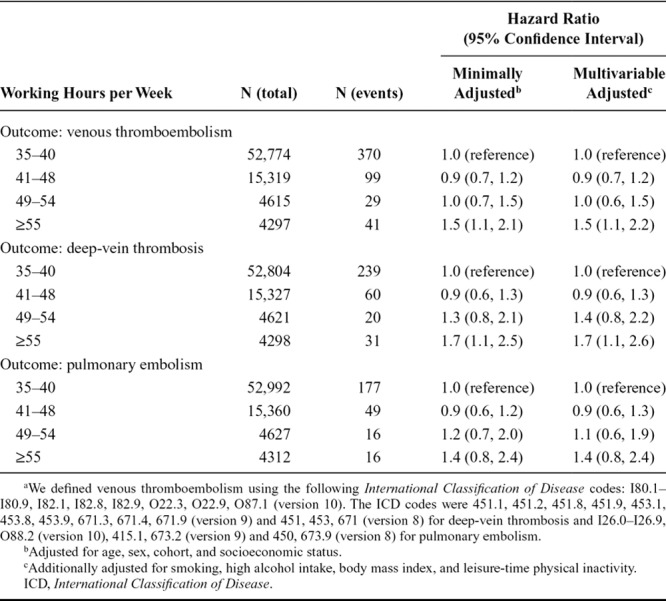
Multivariable-adjusted Association of Weekly Working Hours with Venous Thromboembolism, Deep-Vein Thrombosis and Pulmonary Embolism^a^

In the Table, we show associations between working hours and VTE. The hazard ratio of VTE for individuals working long hours compared with those working standard hours was 1.5 (95% confidence interval [CI] = 1.1, 2.1). The association with deep-vein thrombosis was stronger (hazard ratio = 1.7, 95% CI = 1.1, 2.5), while the association with pulmonary embolism was less robust (hazard ratio = 1.4, 95% CI = 0.8, 2.4). We found no evidence of heterogeneity across studies (*I*^2^ = 0.0%). There was no suggestion that these associations were explained by confounding by common vascular risk factors, including smoking, high alcohol intake, BMI, or leisure-time physical inactivity.

Long working hours have been shown to be associated with increased risk of arrhythmias.^7^ Irregular rhythm—by disrupting the flow of circulation—can cause blood to pool in the left atrial appendage, contributing to clot formation, especially in the presence of hypercoagulability, a condition also underlying VTE.^1,2^ The clot can then travel from the heart to the brain and result in a stroke.^1,2^ In agreement with this link is the observation of increased stroke risk in individuals who work long hours.^6^ The present study completes the picture by reporting an association between long working hours and hypercoagulability on the venous side of the circulation, as indicated by increased risk of VTE, in particularly deep-vein thrombosis.

These results should be viewed with the following limitations in mind. While we took into account a wide array of covariates, including lifestyle variables and occupational group, we did not have data on prior surgery, major trauma, or blood conditions that increase the tendency toward blood clotting, all of which increase the likelihood of VTE.^2^ Lack of adjustment for these characteristics, given that they are linked with reduced rather than increased working hours, may have led to an underestimation of the association with VTE. Unmeasured variation in working hours over time, if random, may also have attenuated observed associations by increasing exposure misclassification. Despite these concerns, our results nonetheless suggest that individuals who work long hours may experience an elevated risk of VTE.

## Supplementary Material

**Mika Kivimäki** Department of Epidemiology and Public Health University College London London, United Kingdom Clinicum, Faculty of Medicine University of Helsinki Helsinki, Finland Finnish Institute of Occupational Health Helsinki, Finland m.kivimaki@ucl.ac.uk**Solja T. Nyberg** Clinicum, Faculty of Medicine University of Helsinki Helsinki, Finland**G. David Batty** Department of Epidemiology and Public Health University College London London, United Kingdom**Ida E. H. Madsen** National Research Centre for the Working Environment Copenhagen, Denmark**Adam G. Tabák;** for the IPD-Work Consortium* Department of Epidemiology and Public Health University College London London, United Kingdom Semmelweis University Faculty of Medicine 1st Department of Medicine Budapest, Hungary
