# Supplementary material for: Long Working Hours and Risk of Venous Thromboembolism
Source: Epidemiology. 2018 Jul 31;29(5):e42–4. doi: 10.1097/EDE.0000000000000862 (PMC6072371; doi:10.1097/EDE.0000000000000862)
Supplement: Supplementary file 1 [file ede-29-e42-s001.pdf]

eAppendix

Participating European, prospective cohort studies with data on long working hours and VTE were:

1. The Finnish Public Sector Study[1] and
2. The Health and Social Support Study,[2] Finland;
3. - 4. The Work, Lipids and Fibrinogen Studies Stockholm[3] and Norrland,[4] Sweden;
5. The Danish Work Environment Cohort Study[5] and
6. - 7. The Copenhagen Psychosocial Questionnaire Studies I and II,[6,7] Denmark;
8. The Whitehall II Study,[8] UK.

All these studies providing individual participant data for our pooled analysis had obtained ethical approval. Baseline assessment varied between 1992 and 2000 depending on the study. Of the 77,291 full-time employees included in this analysis, 48,693 (63%) were women, the mean age of participants at baseline was 43.5 years (study-specific means range: 39.6 and 53.2), and 4,297 (6%) worked long hours. During 830,550 person-years at risk, 539 first-time VTE events were recorded, the incidence being 64.9 per 100,000 person-years.

For comparison, the estimated annual incidence rates of VTE in the general population of European ancestry range from 104 to 183 per 100,000 person-years.[9] The lower incidence of VTE in our study of a working population is as expected given the young mean age and the exclusion of retired people (the incidence of VTE increases with age). In the general population, much of VTE events occur in older people secondary to other health disorders, such as cancer or trauma, or due to treatments, such as recent surgery or immobilization. Known as the healthy worker effect, by definition, working populations do not typically include those with major health conditions.

References

1. Kivimäki M, Lawlor DA, Davey Smith G, et al. Socioeconomic position, co-occurrence of behavior-related risk factors, and coronary heart disease: the Finnish Public Sector study. *Am J Public Health.* 2007;97:874-879.
2. Korkeila K, Suominen S, Ahvenainen J, et al. Non-response and related factors in a nation-wide health survey. *Eur J Epidemiol* 2001;17:991-999.
3. Peter R, Alfredsson L, Hammar N, Siegrist J, Theorell T, Westerholm P. High effort, low reward, and cardiovascular risk factors in employed Swedish men and women: baseline results from the WOLF Study. *J Epidemiol Community Health.* 1998;52:540-547.
4. Alfredsson L, Hammar N, Fransson E, et al. Job strain and major risk factors for coronary heart disease among employed males and females in a Swedish study on work, lipids and fibrinogen. *Scand J Work Environment Health.* 2002; 28: 238-48.
5. Feveile H, Olsen O, Burr H, Bach E. Danish Work Environment Cohort Study 2005: from idea to sampling design. *Stat Transit.* 2007;8:441-458.
6. Kristensen TS, Hannerz H, Hogh A, Borg V. The Copenhagen Psychosocial Questionnaire--a tool for the assessment and improvement of the psychosocial work environment. *Scand J Work Environ Health.* 2005;31:438-449.
7. Pejtersen JH, Kristensen TS, Borg V, Bjorner JB. The second version of the Copenhagen Psychosocial Questionnaire. *Scand J Public Health.* 2010;38(3 Suppl):8-24.
8. Marmot MG, Davey Smith G, Stansfeld S, et al. Health inequalities among British civil servants: the Whitehall II study. *Lancet.* 1991;337:1387-1393.
9. Heit JA. Epidemiology of venous thromboembolism. *Nat Rev Cardiol*. 2015;12: 464–474.

eFigure 1. Flow Chart for Derivation of Analytical Sample

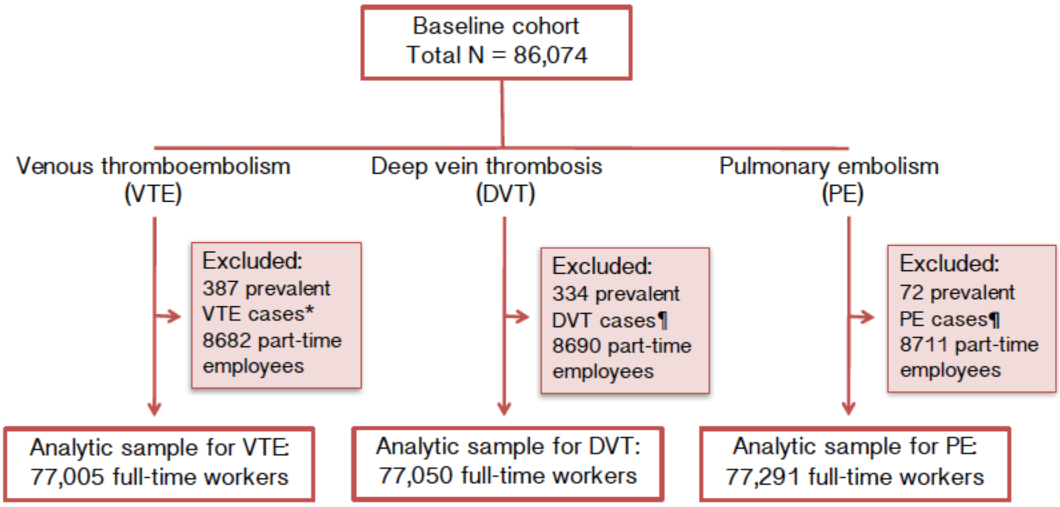

*VTE includes DVT and PE.
¶19 participants had a history of both DVT and PE.
